# Does physical exercise improve or deteriorate treatment of multiple sclerosis with mitoxantrone? Experimental autoimmune encephalomyelitis study in rats

**DOI:** 10.1186/s12868-022-00692-1

**Published:** 2022-03-05

**Authors:** Mohamed A. El-Emam, Samar El Achy, Dalaal M. Abdallah, Hanan S. El-Abhar, Mennatallah A. Gowayed

**Affiliations:** 1 Department of Pharmacology and Therapeutics, Faculty of Pharmacy, Pharos University in Alexandria, Alexandria, Egypt; 2 Department of Pathology, Faculty of Medicine, Alexandria University, Alexandria, Egypt; 3 Department of Pharmacology and Toxicology, Faculty of Pharmacy, Cairo University, Cairo, Egypt; 4 Department of Pharmacology, Toxicology and Biochemistry, Faculty of Pharmacy, Future University in Egypt, New Cairo, Egypt

**Keywords:** Mitoxantrone, Foxp3, Exercise, Demyelination, Anti-inflammatory, Experimental Autoimmune Encephalomyelitis

## Abstract

**Background:**

Mitoxantrone has proved efficacy in treatment of multiple sclerosis (MS). The fact that physical exercise could slow down the progression of disease and improve performance is still a debatable issue, hence; we aimed at studying whether combining mitoxantrone with exercise is of value in the management of MS.

**Methods:**

Thirty-six male rats were divided into sedentary and exercised groups. During a 14-day habituation period rats were subjected to exercise training on a rotarod (30 min/day) before Experimental Autoimmune Encephalomyelitis (EAE) induction and thereafter for 17 consecutive days. On day 13 after induction, EAE groups (exercised &sedentary) were divided into untreated and mitoxantrone treated ones. Disease development was evaluated by motor performance and EAE score. Cerebrospinal fluid (CSF) was used for biochemical analysis. Brain stem and cerebellum were examined histopathological and immunohistochemically.

**Results:**

Exercise training alone did not add a significant value to the studied parameters, except for reducing Foxp3 immunoreactivity in EAE group and caspase-3 in the mitoxantrone treated group. Unexpectedly, exercise worsened the mitoxantrone effect on EAE score, Bcl2 and Bax. Mitoxantrone alone decreased EAE/demyelination/inflammation scores, Foxp3 immunoreactivity, and interleukin-6, while increased the re-myelination marker BDNF without any change in tumor necrosis factor-α. It clearly interrupted the apoptotic pathway in brain stem, but worsened EAE mediated changes of the anti-apoptotic Bcl2 and pro-apoptotic marker Bax in the CSF.

**Conclusions:**

The neuroprotective effect of mitoxantrone was related with remyelination, immunosuppressive and anti-inflammatory potentials. Exercise training did not show added value to mitoxantrone, in contrast, it disrupts the apoptotic pathway.

**Supplementary Information:**

The online version contains supplementary material available at 10.1186/s12868-022-00692-1.

## Background

The cytotoxic, synthetic anthracenedione derivative, mitoxantrone, is an antineoplastic, immunomodulatory agent [1]. It has shown to be one of the most effective agents for treatment of relapsing–remitting, progressive relapsing and secondary progressive multiple sclerosis (MS), having the ability to slow down the worsening of neurological disability [2]. However, its effect on neuropathological hallmarks of the disease have been poorly studied [3], likely due to the arrival of new compounds with a safer profile and a higher patients’ compliance. Being an FDA approved disease-modifying agent for patients with relapsing–remitting MS, it is still being used in countries with economic limits instead of the newer immunomodulatory drugs [4, 5]. Its application is limited mainly due to the cardiotoxicity associated with long-term anthracycline therapy. In cancer patients, the occurrence of cardiotoxicity is expected to be about 3% [6], whereas in MS patients, 3.4% of mitoxantrone recipients had a decrease in left ventricular ejection fraction (LVEF) to ≤ 50% following 1 year of monotherapy and at the end of the second year, relevant incidences were 1.9% [2]. Nonetheless, when used as recommended, the risk of considerable myelosuppressive and cardiotoxic effects decline. Therefore, the lifetime collective dose should be firmly restricted to 140 mg/m^2^ or 2—3 years of therapy and it is not recommended in those with a LVEF of < 50%, those who exhibit a clinically considerable reduction in LVEF throughout treatment, those with a neutrophil count of < 1500 cells/mm3 or those with hepatic impairment [7, 8]. Later, therapy-related acute leukemia in mitoxantrone treated MS patients has also been reported [9, 10].

Exercise training represents a behavioral method for safely managing functional and symptomatic patients, as well as improving their quality of life. It has been found that exercise training can produce small, but important improvements in walking, balance, cognition, fatigue, and depression in MS [11]. Nonetheless, contradictory data has been published on the effects of exercise on molecular pathways in MS patients; while some studies reported a decrease in cytokine levels upon eight weeks of exercise, others reported no effect [12, 13]. In rodents, exercise has shown to increase the release of BDNF, which supports cell proliferation, synaptic plasticity, neuroprotection, and neurogenesis in both physiological and neuroinflammatory conditions [14].

Hence, this study was designed to evaluate the possible influence of exercise with mitoxantrone treatment on the neuronal function and disease progression through an acute relapse of experimental autoimmune encephalomyelitis (EAE).

## Materials and methods

### Animals

A total of 36 male Sprague–Dawley rats (200–250 g, 2–3 months old) were supplied by the animal house of Pharos University in Alexandria (Alexandria, Egypt). Animals had free access to food and water and stayed in air-conditioned room (23 ± 1 °C) with 12 h light–dark cycle. The Research Ethics Committee of the Faculty of Pharmacy, Cairo University approved the study design (Cairo, Egypt, Permit Number: PT 1978). All procedures comply with ARRIVE guidelines, as well as the National Research Council’s guide for the care and use of laboratory animals. Two blinded individuals completed all observations in an arbitrary routine.

### Induction of EAE

Induction proceeded as described previously [15, 16]. Briefly, rats were immunized using a single subcutaneous injection (200 µl) of a mixture of complete Freund’s adjuvant (12 mg/ml) and spinal cord homogenate (50 mg/ml).

### Experimental design

A group of 36 rats were distributed between sedentary (n = 18, SED) and exercised (n = 18, EX) rats. Before disease induction (days -14 to -1) exercised rats were trained for 30 min/day to move on a rotarod as described earlier [15].

**Fig. 1:**
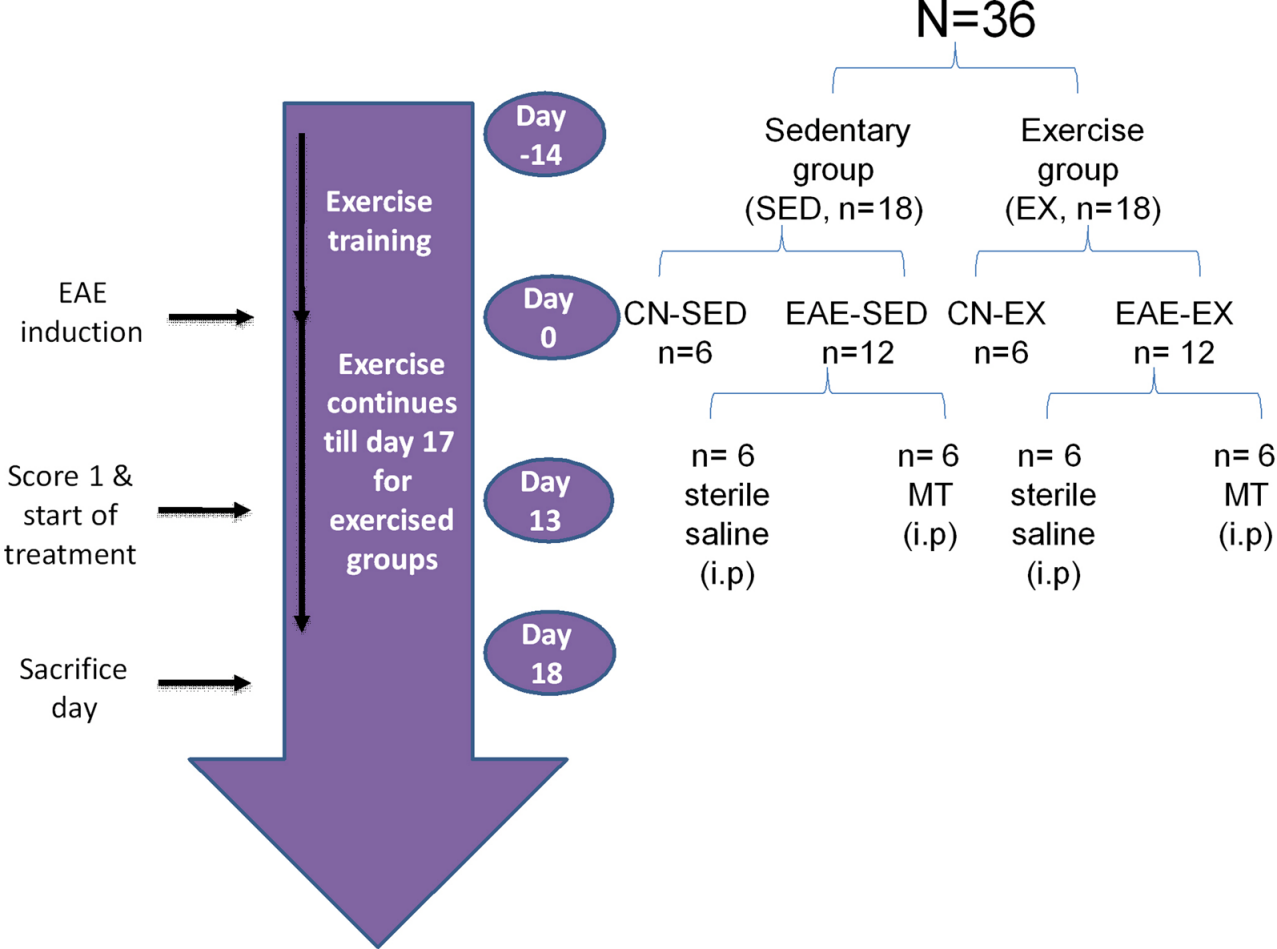
Experimental design timeline. Rats were familiarized to rotarod running before EAE induction (days -14 to -1). After EAE induction (on day 0), control and EAE rats were subjected to daily physical exercise for 17 days. Upon appearance of EAE symptoms (score 1, on day 13) rats were subjected to treatment with mitoxantrone (MT). Finally, all rats were sacrificed on day 18. *EAE* experimental autoimmune encephalomyelitis, *CN*_*SED*_ sedentary control, *CN*_*EX*_ exercised control; EAE_SED_ = sedentary untreated EAE; EAE_EX_ = exercised untreated EAE rats

On day 0, EX and SED rats were divided into control (CN_SED_; CN_EX_, n = 6) and untreated EAE (EAE_SED_; EAE_EX_, n = 12) rats. After induction of EAE, exercised rats maintained their daily rotarod training (20 rpm) till day 17 post EAE induction. On day 13, when all rats developed score 1 of EAE, the EAE (EAE_SED_ & EAE_EX_) rats were divided into four groups (n = 6). Groups I and II were control sedentary (CN_SED_) and exercised (CN_EX_) rats. Groups III & IV were untreated EAE rats (EAE_SED_ & EAE_EX_) receiving saline (i.p). Groups V & VI were injected daily with mitoxantrone (MT, 2.5 mg/kg/day; i.p; Santrone©; EIMC United Pharmaceuticals; Alexandria, Egypt) and identified as mitoxantrone sedentary (MT_SED_) & mitoxantrone exercised (MT_EX_) [17, 18] starting on day 13 after induction and rats were given the mitoxantrone dose 1 h before the training. Figure 1 shows the experimental outline of the study.

After EAE induction, the animal’s overall health has been observed for any changes. Rats were treated for 5 days, from day 13 till the end of the experiment (day 17). Drug treatment was given 1 h before exercise [19].

### Assessment of MS progression

#### EAE score

After EAE induction, rats were monitored daily for the EAE score, as indicator of disease progression and muscular tone. The score was evaluated as described previously [20] and illustrated in Table 1.

**Table 1:** EAE scoring system [20]

EAE score	
0	No sign
1	Flaccid tail
2	Weakness of one hind limb
3	Weakness of the two hind limbs/walking difficulties
4	Paraplegia
5	Moribund/death

#### Rotarod test

Before EAE induction (days-14 to -1) only rats in the EX-groups were exercised to stay on the rotarod for 30 min (20 rpm). After EAE induction, rats continued to be trained on the metallic rod daily till the end of the experiment (days 0 to 17). The falling time was agreed as indicator of motor performance [19].

#### Sample processing

On day 18 after EAE induction, rats were euthanized with an overdose of phenobarbital (100 mg/kg). Samples were then collected for further biochemical, histological and immunohistochemistry studies. Rats were then kept frozen till incineration.

The cerebrospinal fluid (CSF) was used to examine the amounts of tumor necrosis factor-α (TNF-α) [CAT# BEK1214] and interleukin-6 (IL-6) [CAT# BEK1110], BDNF as re-myelination indicator (CAT# BEK1013), the apoptosis related markers Bcl-2 [CAT# CSB-E08854r] and Bax [CAT# CSB-EL002573RA]. The ELISA kits were purchased from Chongqing Biospes (Chongqing, PRC) and Cusabio Technology LLC (TX, USA) and used according to manufacturers' instructions.

#### Histopathological study

The brain stem and cerebellum were separated, washed with phosphate buffered saline (PBS) and immersed in 10% formaldehyde to be processed into paraffin blocks. Samples were then sliced into 5 μm thickness, mounted on glass slides, then stained using hematoxylin and eosin (H&E) to evaluate morphological changes between the different groups. Scoring of inflammation was performed as described previously and illustrated in Table 2 [21].

**Table 2:** Scoring system of inflammation and demyelination [21]

Inflammatory score	
0	No inflammatory cells
1	Few scattered inflammatory cells
2	Organization of inflammatory infiltrates around blood vessels
3	Extensive perivascular cuffing with extension into adjacent parenchyma
Demyelination score	
0	Normal
1	One small focal area of demyelination
2	Two or three areas of demyelination
3	One to two large areas of demyelination
4	Extensive demyelination involving ≥ 20% of the white matter

Luxol fast blue (LFB) staining was used for assessment of demyelination in the cerebellum and brain stem and scored as described previously by Zhang et al*.* [21] and illustrated in Table 2. Histopathological examination was performed and scored in a blinded fashion.

### Immunohistochemistry

For the immunohistochemical staining, 5 µm thick sections were fixed onto positively charged slides, deparaffinized and hydrated in xylene and descending alcohol solutions, and finally rinsed with PBS. Citrate buffer (pH 6.0) was used for heat induced antigen retrieval. Endogenous peroxidase was blocked with H_2_O_2_ and endogenous biotin with the aid of a blocking Kit Avidin/Biotin (DAKO #X0909, Glostrup, Denmark). After incubation in blocking buffer, the sections were treated with primary antibodies against Caspase-3 (Cell Signaling Technology, MA, USA, CAT#9662) with a dilution of 1:1000, and Foxp3 (FOXP3 Monoclonal Antibody, Invitrogen, Thermo Fisher Scientific, CA, USA, CAT# 14-5773-82) with a dilution of 5 µg/ml. Reactions were visualized using EnVision + System-HRP Kit (DAKO #K4063, Glostrup, Denmark). DAB (DAKO #K4063 Glostrup, Denmark) was used as chromogen and the tissue was counterstained with Mayer’s Hematoxylin. The number of positive cells in hotspot areas in ten high power fields (HPFs) in areas of demyelination and plaques in the brain stem were counted using the image analysis software (Leica Application Suite Version 4.12.0, Wetzlar, Germany).

### Statistical analysis

Data are showed as mean ± standard deviation (S.D). For parametric data, one-way analysis of variance (ANOVA) was used, followed by Tukey’s Multiple Comparisons Test as post hoc. Interaction of exercise and treatment for dependent variables was analyzed using two-way ANOVA, followed by Bonferroni Correction Test. The EAE and histopathology scores were analyzed using Kruskal–Wallis Test, followed by Dunn's post hoc test, and presented as median with range. *P* < *0.05* was considered the significance limit for all comparisons. All analysis and graphs were performed using Prism computer program (GraphPad software Inc. V5, CA, USA).

## Results

### General Observation of animal health

**Fig. 2:**
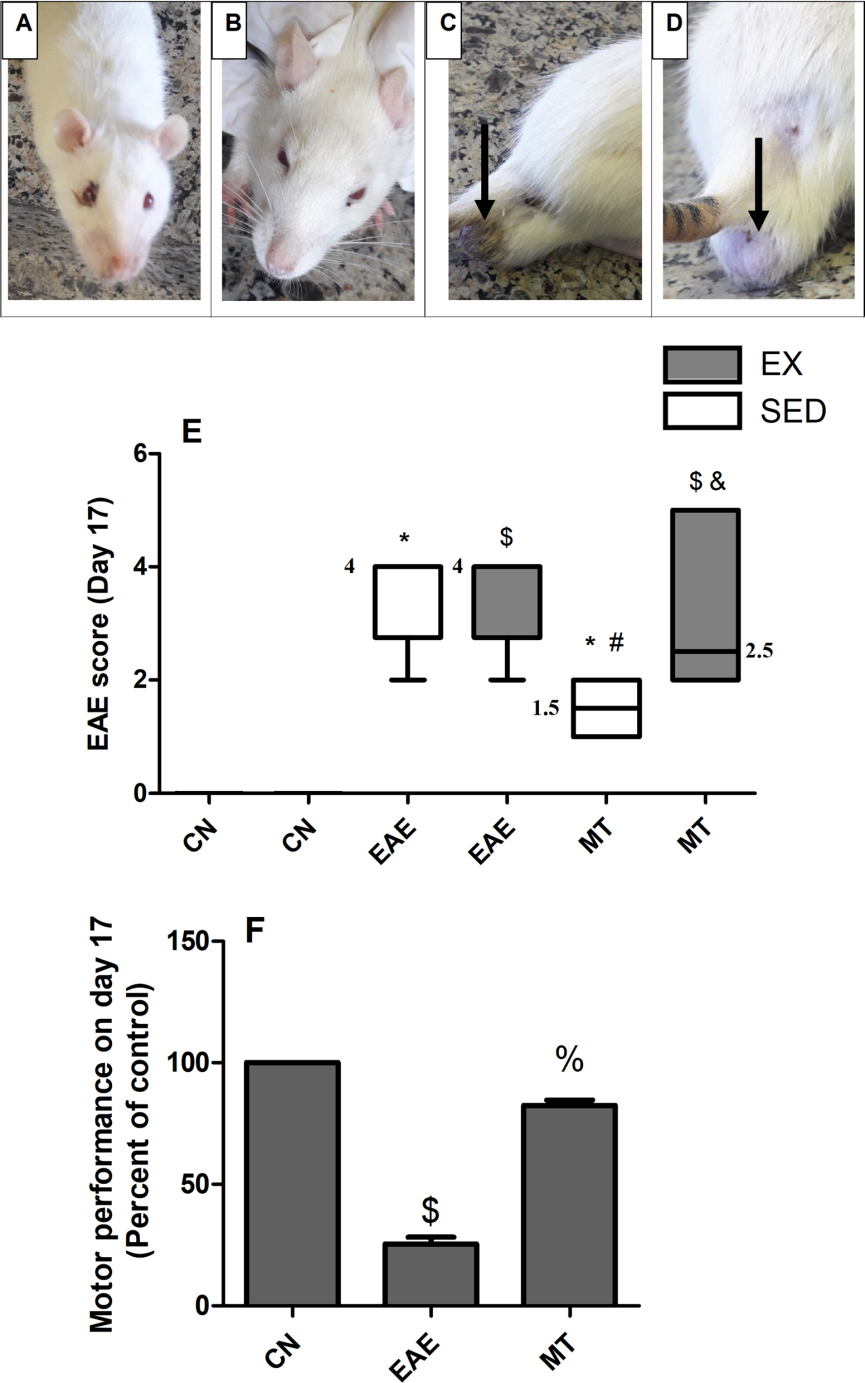
Effect of exercise on the animal health, the EAE score and motor performance. **A** shows representative photographs of mitoxantrone treated rats on day 17 showing porphyrin spots near the eye of exercised and **B** sedentary rats, as well as **C** and **D** blue discoloration of their testes was noted. **E** represents the EAE score. Data were analyzed using Kruskal–Wallis Test, followed by Dunn's post hoc test and presented as median values with range, *p* < 0.05. The median values are presented as CN_SED_ = 0, CN_EX_ = 0, EAE_SED_ = 4, EAE_EX_ = 4, MT_SED_ = 1.5 and MT_EX_ = 2.5. **F** shows the percentage to control of motor performance on day 17. Data were analyzed using one-way ANOVA followed by Tukey's Multiple Comparison Test and presented as mean ± S.D (*p* < 0.05). As compared with CN_SED_ (*), CN_EX_ ($), EAE_SED_ (#), EAE_EX_ (%) and MT_SED_ (&) groups. *EAE* experimental autoimmune encephalomyelitis; groups of *CN*_*SED*_ sedentary control, *CN*_*EX*_ exercised control, *EAE*_*SED*_ sedentary untreated EAE; *EAE*_*EX*_ exercised untreated EAE; *MT*_*SED*_ sedentary mitoxantrone, *MT*_*EX*_ exercised mitoxantrone; (n = 6/group)

The overall health status of sedentary and exercised rats treated with mitoxantrone was declining throughout the study; however, no change in weight was observed. Additionally, and as depicted in Fig. 2A, B porphyrin spots near the eye, as well as (C, D) blue discoloration of testis was observed in the mitoxantrone groups.

### Effect of mitoxantrone on EAE score

As depicted from Table 3, on day 13 after EAE induction, all rats attained score 1. On day 14, untreated rats (EAE_SED_ and EAE_EX_) weakened reaching score 2, while treated rats stayed at score 1. There was a fluctuation in the scores among groups for the 5 days of treatment till day 17. On day 17, the EAE_EX_ and EAE_SED_ rats deteriorated to reach a median score 4. The sedentary rats treated with mitoxantrone had a median score of 1.5; whereas, exercise worsened the case and elevated the median score to 2.5 in the MT_EX_ group to be insignificant from the untreated animals. Figure 2E shows the median (min–max) scores on day 17 post EAE.

**Table 3:** Effect of mitoxantrone on EAE score in sedentary and exercised EAE rats (day 13–day 17)

Parameter	EAE score				
Groups	Day 13	Day 14	Day 15	Day 16	Day 17
CN_SED_	0(0–0)	0(0–0)	0(0–0)	0(0–0)	0(0–0)
CN_EX_	0(0–0)	0(0–0)	0(0–0)	0(0–0)	0(0–0)
EAE_SED_	1(1–1)	2(2–2)	2(2–3)	2(2–3)	4(2–4)^*^
EAE_EX_	1(1–1)	2(2–2)	2(2–2)	3(2–3)	4(2–4)^$^
MT_SED_	1(1–1)	1(1–2)	2(2–2)	2(2–3)	1.5(1–2)^*#^
MT_EX_	1(1–1)	1(1–1)	2(1–2)	2(2–5)	2.5(2–5) ^$&^

Non-parametric score data are shown as median with its minimum and maximum range (n = 6). Comparisons between groups at each time interval was analyzed using Kruskal–Wallis Test, followed by Dunn's post hoc test, *p* < *0.05*. As compared with (*) CN_SED_, ($) CN_EX_, (#) EAE_SED_ and (&) MT_SED_ groups. *EAE* experimental autoimmune encephalomyelitis, *CN*_*SED*_ sedentary control, *CN*_*EX*_ exercised control; EAE_SED_: sedentary untreated EAE, *EAE*_*EX*_ exercised untreated EAE, *MT*_*SED*_ sedentary mitoxantrone, *MT*_*EX*_ exercised mitoxantrone treated rats

### Effect of mitoxantrone on motor performance

During the training period (days-14 to -1), a gradual increase in the motor performance of all EX-rats was observed until reaching a steady state of 1800s on day -9 (before EAE induction; Additional file 1) and continued till day 13 after EAE induction. Table 4 presents the changes in motor performance of EAE_EX_ rats. On day 13, an obvious discrepancy was observed between control EX rats and EAE exercised ones. Interestingly, EX animals treated with mitoxantrone have shown fluctuation in motor function till day 15 being almost similar to EX-EAE rats, an effect that vanished later showing a gradual recovery afterwards till reaching almost same values as CN_EX_ on day 17 (Fig. 2F).

**Table 4:** Effect of mitoxantrone on motor performance of exercised EAE rats (day 13—day 17)

Parameter	Motor performance (s)				
Groups	Day 13	Day 14	Day 15	Day 16	Day 17
CN	1800 ± 0	1800 ± 0	1800 ± 0	1800 ± 0	1800 ± 0
EAE	711 ± 215.5^$^	625.4 ± 190.2^$^	599.2 ± 183.0^$^	540.8 ± 165.9^$^	453.3 ± 143.5^$^
MT	711 ± 215.5^$^	950.7 ± 380.2	664.4 ± 362.0^$^	1028 ± 350.4	1500 ± 300.0^%^

Values are presented as mean ± S.D (n = 6) and were analyzed using one-way ANOVA followed by Tukey's Multiple Comparison Test (*p* < 0.05). As compared with ($) CN and (%) EAE groups. *EAE* experimental autoimmune encephalomyelitis, *CN* exercised control, *EAE* exercised untreated EAE, *MT* exercised mitoxantrone treated rats

### Effect of mitoxantrone on histopathological changes in the brain

**Fig. 3:**
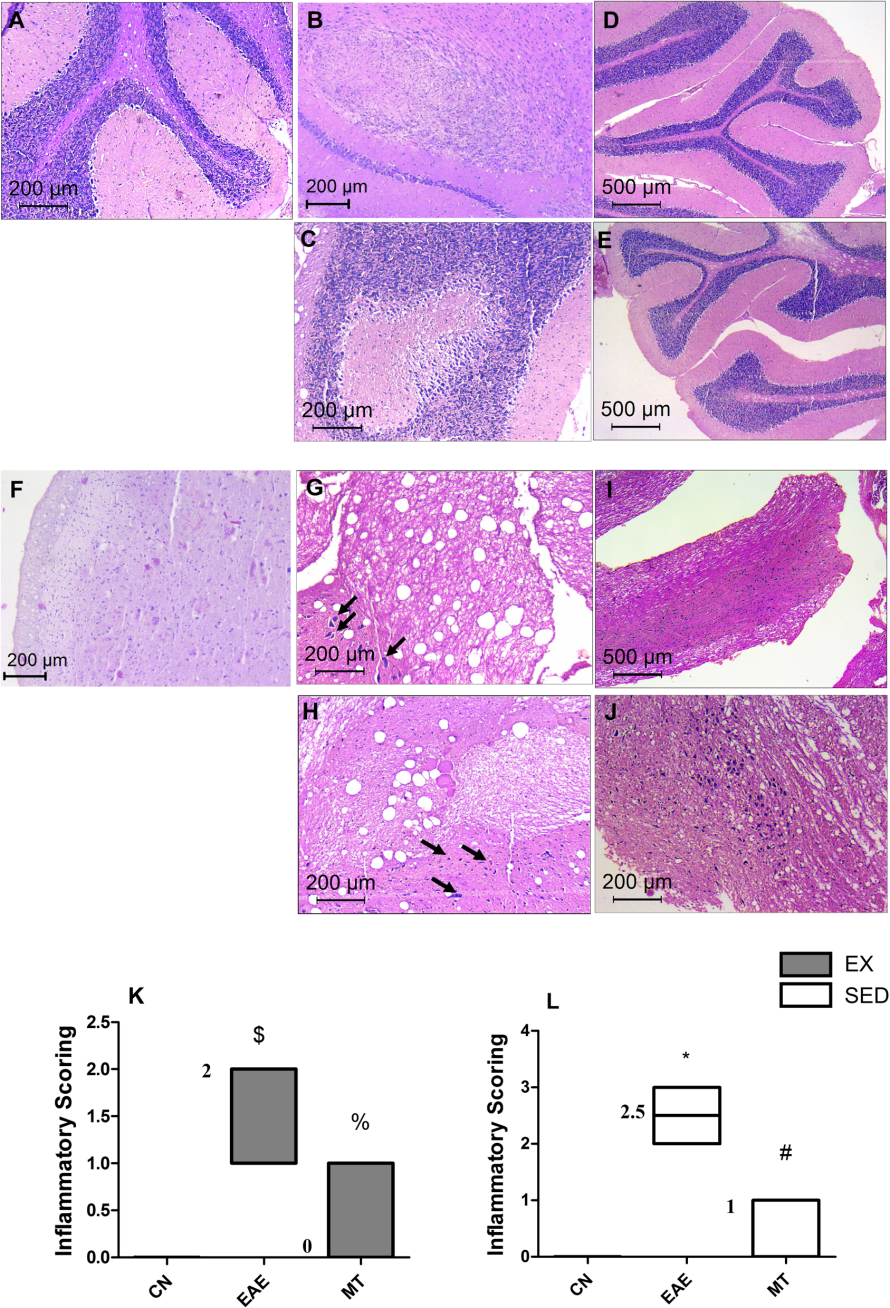
Histopathological examination of rats’ brain treated with mitoxantrone. **A** Photomicrograph of control rat cerebellum showing no inflammation and no demyelinating plaques, while photomicrographs of EAE **B** SED and **C** EX groups show leukocortical plaques with inflammatory infiltration and decreased neurons in the granular cell layer. Sections of mitoxantrone treated **D** EAE sedentary and **E** exercised groups reveal reduced inflammatory cellular infiltrates, preservation of the white matter and grey matter, as well as near complete healing of the plaques. Compared to **F** normal control brain stem, sections of **G** sedentary and **H** exercised EAE groups show an active plaque with reduced numbers of axons and microcyst formation. Reactive astrocytes are surrounding the plaque (black arrows) and lymphocytic infiltrates. Sections of mitoxantrone treated EAE **I** sedentary and **J** exercised group reveal reduced inflammatory cellular infiltrates and increased axons with reduced microcyst formation. Reactive astrocytes and gliosis are more pronounced at the periphery. **K** and **L** summarize inflammation scores in the cerebellum and brain stem among different groups in exercised and sedentary rats, respectively. Non-parametric data were analyzed using Kruskal–Wallis Test followed by Dunn's Multiple Comparison Test and presented as median with range (p < 0.05). As compared with CN_SED_ (*), CN_EX_ ($), EAE_SED_ (#) and EAE_EX_ (%). The median values are presented as CN_SED_ = 0, CN_EX_ = 0, EAE_SED_ = 2.5, EAE_EX_ = 2, MT_SED_ = 1 and MT_EX_ = 0. *EAE* experimental autoimmune encephalomyelitis; groups of CN_SED_ = sedentary control; *CN*_*EX*_ exercised control, *EAE*_*SED*_ sedentary untreated EAE, *EAE*_*EX*_ exercised untreated EAE, *MT*_*SED*_ sedentary mitoxantrone, *MT*_*EX*_ exercised mitoxantrone; (n = 6/group)

Histopathological examination (Fig. 3) of the (A–C) cerebellum and (F–H) brain stem of untreated EAE groups showed plaques compared to normal control. Active plaques revealed inflammatory cellular infiltrates with abundant macrophages stuffed with myelin debris, an evidence of ongoing myelin breakdown. Lymphocytic infiltrates were also present, mostly as perivascular cuffs. Small active lesions were often seen centered on small veins and axons were relatively preserved, but reduced in number with microcyst formation. Gliosis and reactive astrocytes were surrounding the plaques. Cerebellar plaques were seen in three areas, leukocortical, intracortical and subpial. Leukocortical plaques showed decrease in the Purkinje cell mass inside the lesions. Brain stem plaques were mainly leukocortical in location.

**Fig. 4:**
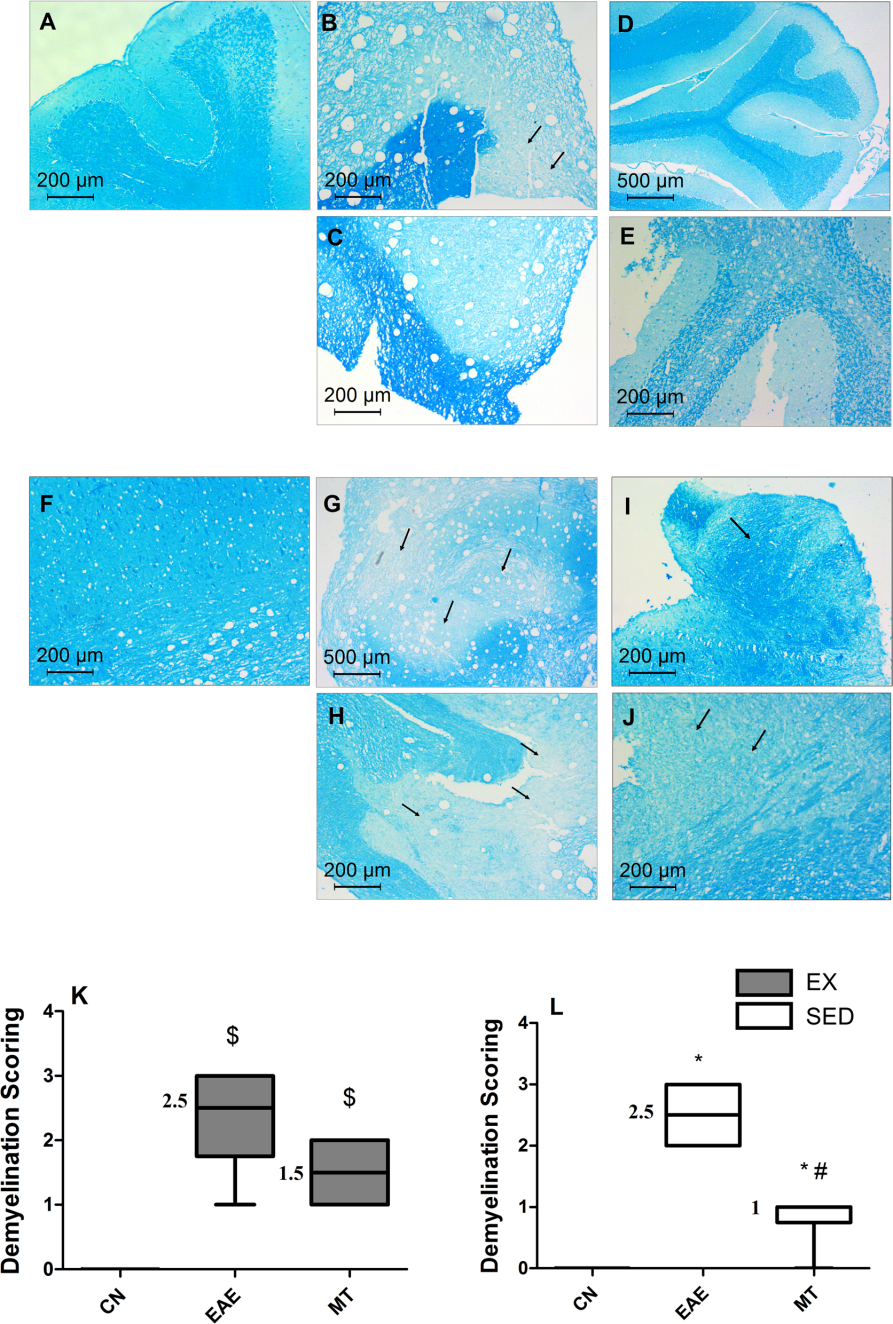
Effect of mitoxantrone with/without exercise on myelination using luxol fast blue staining. Relative to cerebellum section of **A** normal control, **B** EAE sedentary, **C** EAE exercised untreated groups show light spots of demyelination (black arrows). However, sections of mitoxantrone treated **D** sedentary/**E** exercised groups show remyelinated white matter with no evidence of plaques. Similarly, brain stem sections of **G** EAE sedentary/**H** exercised groups present pale spots of demyelination (black arrows) compared to **F** normal control ones. However, sections of mitoxantrone treated **I** sedentary and **J** exercised groups show remyelination of plaques (black arrows). The demyelination scores in the cerebellum and brain stem of exercised and sedentary groups are summarized in panels **K** and **L**, respectively. Non-parametric data were analyzed using Kruskal–Wallis Test followed by Dunn's Multiple Comparison Test and presented as median values with range (p < 0.05). As compared with CN_SED_ (*), CN_EX_ ($), EAE_SED_ (#) and EAE_EX_ (%).The median values are presented as CN_SED_ = 0, CN_EX_ = 0, EAE_SED_ = 2.5, EAE_EX_ = 2.5, MT_SED_ = 1 and MT_EX_ = 1.5. *EAE* experimental autoimmune encephalomyelitis; groups of CN_SED_ = sedentary control; *CN*_*EX*_ exercised control, *EAE*_*SED*_ sedentary untreated EAE; EAE_EX_ = exercised untreated EAE, *MT*_*SED*_ sedentary mitoxantrone, *MT*_*EX*_ exercised mitoxantrone; (n = 6/group)

Inflammatory infiltration was evaluated and scored revealing severe perivascular cuffing in the untreated group. Figure 3K, L show heavy inflammatory cellular infiltrates in the untreated EAE groups. Inflammatory scores in the EAE group were significantly higher than all other treated groups (*p* < 0.0001) reaching a median score of 2 in exercised *versus* 2.5 in sedentary rats. In Fig. 4, the demyelination of (A-C) the cerebellum and (F–H) brain stem was evaluated using LFB staining and median scored 2.5 in both sedentary and exercised untreated EAE rats indicating extensive demyelination.

Post-administration of mitoxantrone to sedentary and exercised groups smaller patches of demyelination with microcyst formation and lymphocytic infiltrates were seen; and bare unmyelinated axons were fewer (Figs. 3D, E, I, J and 4D, E, I, J). These results were reflected on the inflammatory (Fig. 3K, L) and demyelination (Fig. 4K, L) scores in sedentary and exercised rats, reaching median inflammatory scores of 0 in exercised *versus* 1 in sedentary rats with median demyelination scores of 1.5 in exercised rats *versus* 1 in sedentary rats.

### Effect of mitoxantrone on the CSF cytokine levels

**Fig. 5:**
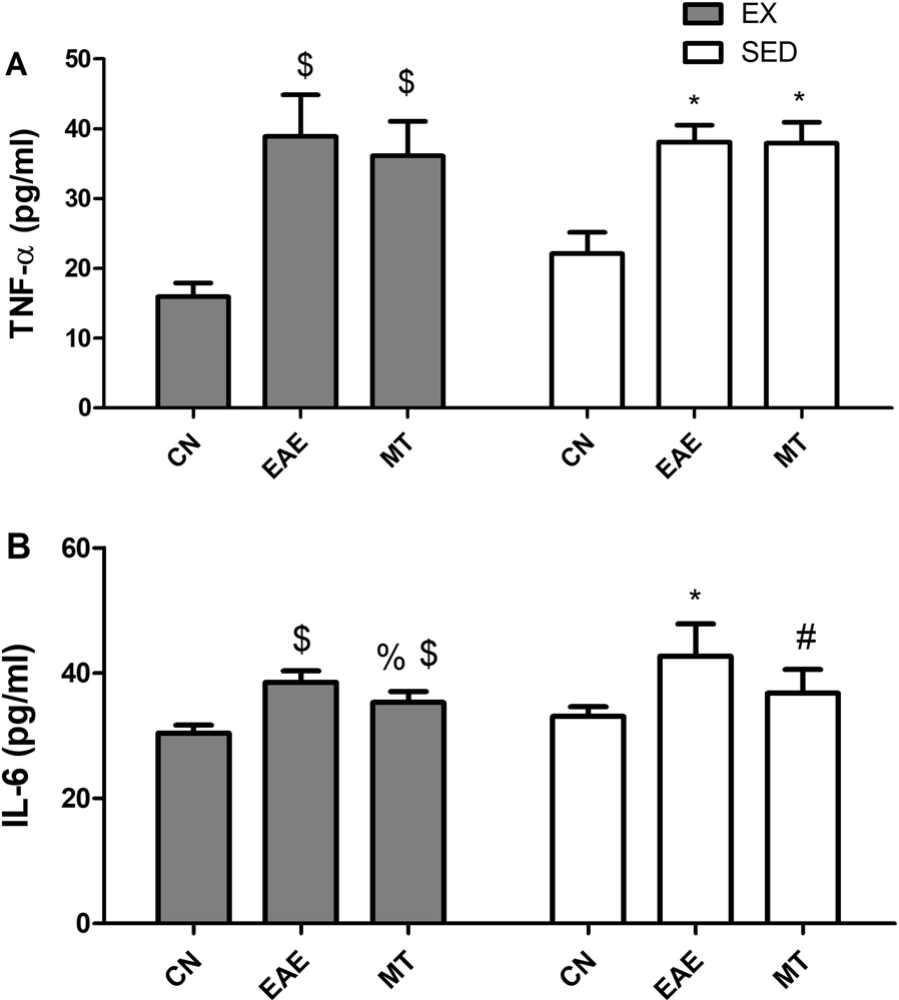
Effect of mitoxantrone with/without exercise on cytokines levels in the CSF. **A** TNF-α, **B** IL-6. Values are presented as mean ± S.D (n = 6/group). Comparison inside the same group was done using one-way ANOVA followed by Tukey's Multiple Comparison Test. Comparison between sedentary and exercised groups was done using two-way ANOVA followed by Bonferroni Correction Test (p < 0.05). As compared to CN_SED_ (*), CN_EX_ ($), EAE_SED_ (#) and EAE_EX_ (%). *EAE* experimental autoimmune encephalomyelitis; groups of CN_SED_ = sedentary control, *CN*_*EX*_ exercised control, *EAE*_*SED*_ sedentary untreated EAE, *EAE*_*EX*_ exercised untreated EAE, *MT*_*SED*_ sedentary mitoxantrone, *MT*_*EX*_ exercised mitoxantrone

EAE induction stimulated the inflammatory cascade (Fig. 5), showing increase in (A) TNF-α and (B) IL-6 levels. Treatment with mitoxantrone could not cause a decline in the TNF-α level whether in sedentary (*p* = 0.8859) or exercised (*p* = 0.4023) groups. In contrast, treatment with mitoxantrone showed a decline in IL-6 levels compared to untreated EAE exercised (*p* = 0.0099) and sedentary rats (*p* = 0.0479). On the other hand, exercise training with mitoxantrone treatment did not show any improvement over its sedentary counterpart (TNF-α *p* = 0.4747 and IL-6 *p* = 0.3962). Two-way ANOVA analysis did not reveal any interaction between drug treatment and exercise on TNF-α (F = 2.51, *p* = 0.0984) nor IL-6 (F = 0.62, *p* = 0.5438).

### Effect of mitoxantrone on BDNF level in the CSF

**Fig. 6:**
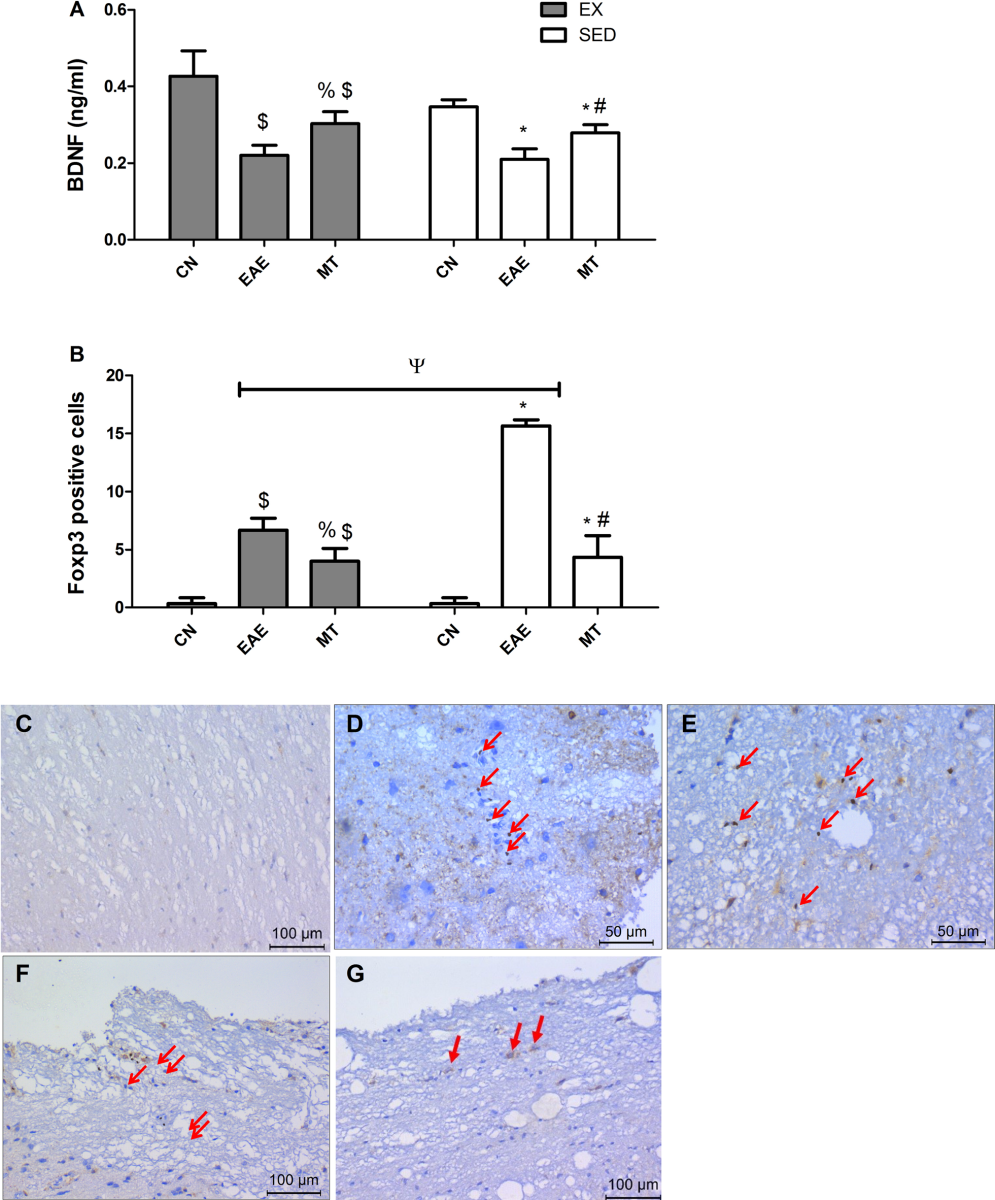
Effect of mitoxantrone with/without exercise on **A** BDNF in the CSF and **B**–**G** Foxp3 in the brain stem of EAE rats. **B** Number of Foxp3 positive cells in the brain stem of exercised and sedentary rats. Immunostaining of Foxp3 showing absence of positively stained lymphocytes in **C** normal control rats. Sections of EAE untreated rats show positively stained perivascular lymphocytes (arrows) in the active plaques of **D** sedentary/**E** exercised groups, whereas treatment with mitoxantrone shows few positively stained lymphocytes in the active plaques of **F** sedentary/**G** exercised groups. Values are presented as mean ± S.D (n = 6/group). Comparisons inside the same group was done using one-way ANOVA followed by Tukey's Multiple Comparison Test*.* Comparison between exercised and sedentary groups was done using two-way ANOVA followed by Bonferroni Correction Test (p < 0.05). As compared to CN_SED_ (*), CN_EX_ ($), EAE_SED_ (#), EAE_EX_ (%) and exercised *vs* sedentary rats (ψ). *EAE* experimental autoimmune encephalomyelitis; groups of *CN*_*SED*_ sedentary control, *CN*_*EX*_ exercised control, *EAE*_*SED*_ sedentary untreated EAE, *EAE*_*EX*_ exercised untreated EAE, *MT*_*SED*_ sedentary mitoxantrone, *MT*_*EX*_ exercised mitoxantrone

As depicted in Fig. 6, the induction of EAE nearly halved (A) the BDNF level in the exercised and sedentary rat groups, compared to their normal groups; however, treatment with mitoxantrone revealed a slight, yet expressive increase in the CSF- BDNF levels compared to untreated control (25% MT_SED_ *versus* 27% MT_EX_). Again, mitoxantrone treatment accompanied with exercise did not add value to the treatment protocol (*p* = 0.1479). Two-way ANOVA analysis did not reveal an interaction between drug treatment and exercise on the BDNF (F = 3.245, *p* = 0.0626).

### Effect of mitoxantrone on Foxp3 in brain stem

An indicator of Treg-cell activity is Foxp3. As shown in Fig. 6, panel (B) summarizes the effect of mitoxantrone with and without exercise on the expression of Foxp3 in the brain stem of untreated exercised and sedentary rats. The photomicrographs, show a marked increase in Foxp3 expression in (D) SED/(E) EX rats as compared to (C) control with a more prominent increase in the sedentary group. On the other hand, mitoxantrone post-administration succeeded in reducing Foxp3 protein expression in (F, G) both treated groups. Exercise did not cause any decrease in the Foxp3 count in rats treated with mitoxantrone, compared to its sedentary counterpart (*p* = 0.7918). Two-way ANOVA analysis revealed interaction between training and treatment (F = 71.79, *p* < 0.0001), which is reflected on the EAE untreated exercised rats.

### Effect of mitoxantrone on caspase-3 in brain stem

**Fig. 7:**
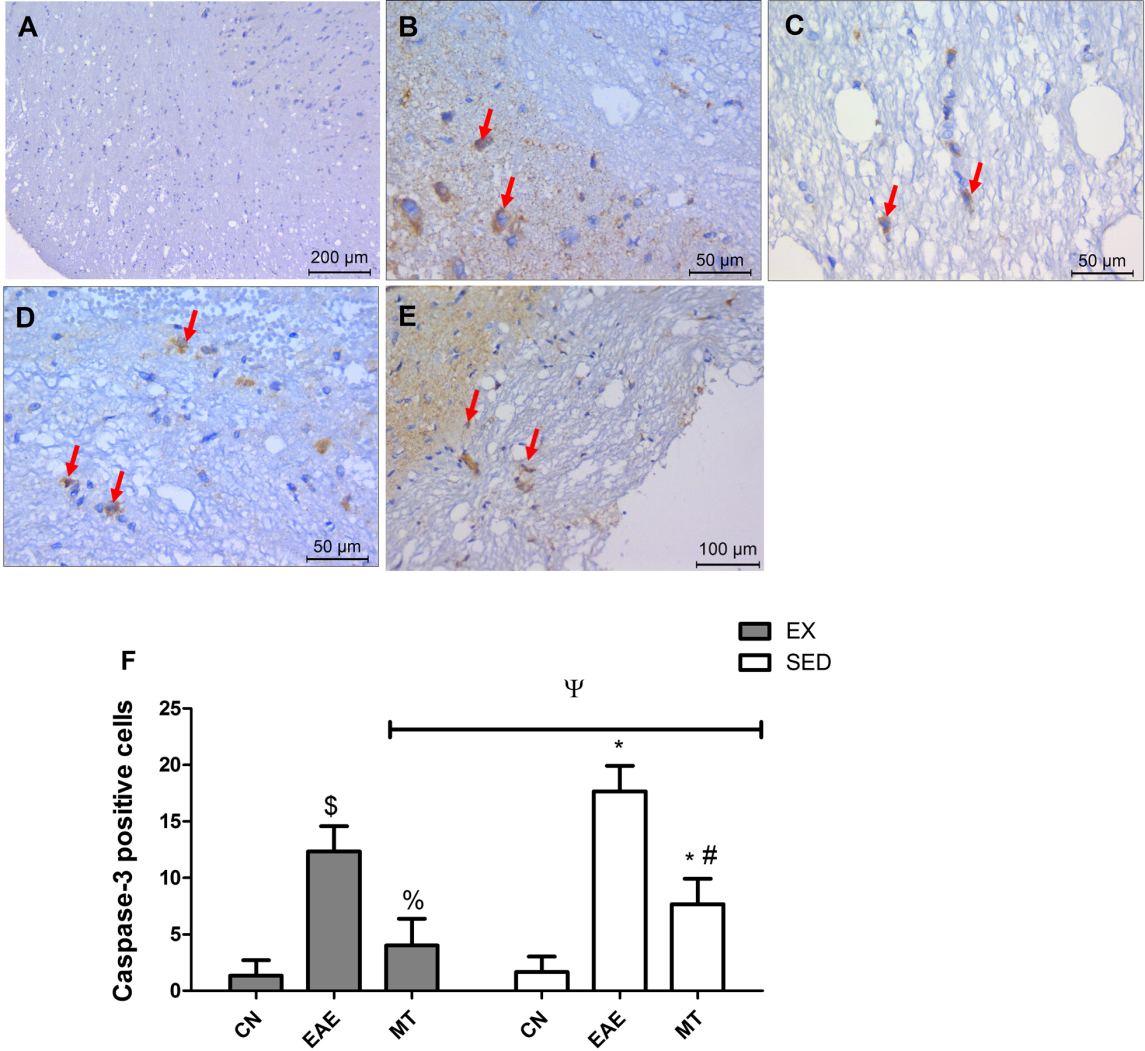
Effect of mitoxantrone on activated caspase-3 in the brain stem. Compared to the negative immunostaining of caspase 3 in **A** normal control group, sections of EAE untreated rat show positive staining in the astrocytes and neurons surrounding the plaques (arrows) of **B** sedentary/**C** exercised groups. Sections of mitoxantrone treated group show few positively stained astrocytes and neurons in the plaques of **D** sedentary/**E** exercised rats. Panel **F** shows the influence of exercise on the amount of caspase-3 positive cells in brain stem. Values are presented as mean ± S.D. (n = 6/group). Comparison inside the same group was done using one-way ANOVA followed by Tukey's Multiple Comparison Test*.* Comparison between sedentary and exercised groups was done using two-way ANOVA followed by Bonferroni Correction Test (p < 0.05). As compared with CN_SED_ (*), CN_EX_ ($), EAE_SED_ (#), EAE_EX_ (%) and exercised *vs* sedentary rats (ψ). *EAE* experimental autoimmune encephalomyelitis; groups of *CN*_*SED*_ sedentary control, *CN*_*EX*_ exercised control, *EAE*_*SED*_ sedentary untreated EAE, *EAE*_*EX*_ exercised untreated EAE, *MT*_*SED*_ sedentary mitoxantrone, *MT*_*EX*_ exercised mitoxantrone

The capase-3 expression (Fig. 7) comes to show the prominent apoptosis in EAE sedentary and exercised groups, where (B) SED and (C) EX rats have shown increased caspase-3 expression, compared to control (A) rats (*p* < 0.0001). However, mitoxantrone treatment has reduced the caspase-3 expression in both (D) SED and (E) EX rats (*p* < 0.0001). Panel (F) recapitulates the elevated caspase-3 expression in EAE rats and the anti-apoptotic capability of post-treatment with mitoxantrone. Exercise clearly enhanced the effect of mitoxantrone compared to its sedentary counterpart (*p* = 0.0205), showing no difference from exercised control (*p* = 0.0758). This effect induced by exercise and treatment is due to the interaction between both factors as shown using two-way ANOVA analysis (F = 4.766, *p* = 0.0159).

### Effect of mitoxantrone on Bcl-2 and Bax levels in the CSF

**Fig. 8:**
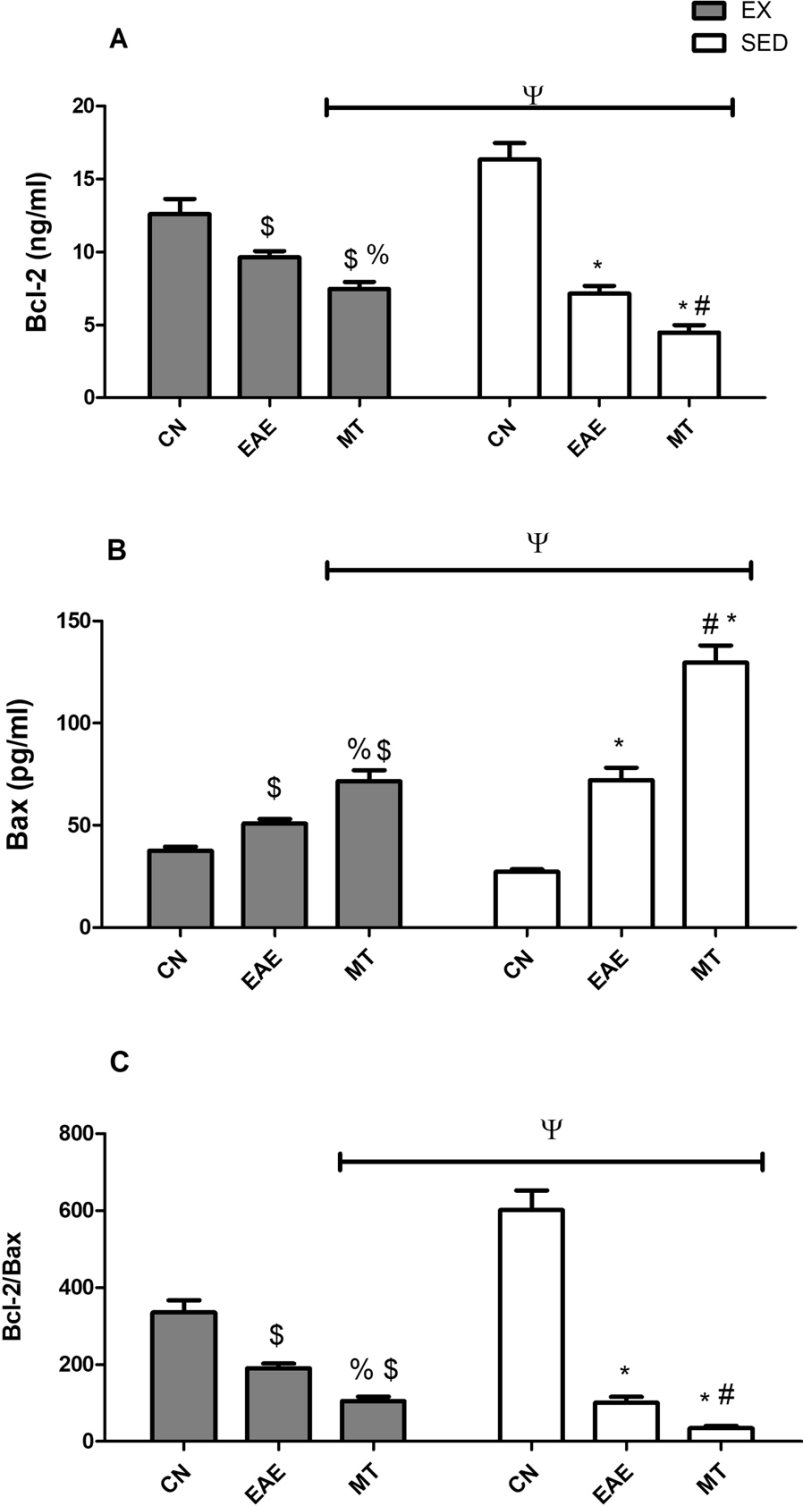
Effect of mitoxantrone with/without exercise on CSF levels of **A** Bcl-2, **B** Bax and **C** Bcl-2/Bax ratio. Values are presented as mean ± S.D. (n = 6). Comparison inside the same group was done using one-way ANOVA followed by Tukey's Multiple Comparison Test. Comparison between sedentary and exercised groups was done using two-way ANOVA followed by Bonferroni Correction Test (p < 0.05). As compared with CN_SED_ (*), CN_EX_ ($), EAE_SED_ (#), EAE_EX_ (%) and exercised *vs* sedentary rats (ψ). *EAE* experimental autoimmune encephalomyelitis; groups of CN_SED_ sedentary control, *CN*_*EX*_ exercised control, *EAE*_*SED*_ sedentary untreated EAE, *EAE*_*EX*_ exercised untreated EAE, *MT*_*SED*_ sedentary mitoxantrone, *MT*_*EX*_ exercised mitoxantrone

Figure 8 comes to confirm the EAE-induced apoptosis, where it showed a significant inhibition in the anti-apoptotic parameter (A) Bcl-2 and an elevation in the apoptotic marker (B) Bax in both untreated EAE sedentary and exercised rats; these effects were less evident in the exercised group. The (C) Bcl-2/Bax ratio in SED and EX groups confirmed the latter observation. Post-treatment with mitoxantrone has worsened the effect of EAE on the tested markers in the SED and EX groups, showing a decrease in Bcl-2 level (*p* = 0.0010 and *p* < 0.0001 respectively), sharp increase in Bax (44% sedentary *versus* 29% exercised), and hence a decrease in Bcl-2/Bax ratio for both groups (*p* < 0.001). Exercise made a subtle, yet significant improvement in the mitoxantrone treated groups compared to its SED counterpart (*p* < 0.0001), where two-way ANOVA shows interaction between mitoxantrone treatment and training (F = 77.7, *p* < 0.0001 for Bcl-2, F = 146.0, *p* < 0.0001 for Bax and F = 171.0, *p* < 0.0001 for Bcl-2/Bax).

## Discussion

Our study has emphasized the influence of exercise with mitoxantrone treatment on the neuronal function and disease management during an EAE animal model. Based on previous findings, exercise has shown effectiveness in MS patients, delaying the progression of disease and restoring the overall health status [22, 23]. Mitoxantrone treatment in doses ranging from 0.2 to 5 mg/kg, given before the onset of clinical signs, has previously shown the ability to inhibit the course of the disease significantly in an EAE model, the most commonly used animal model to study immunopathological mechanisms in MS [1, 17, 24, 25].

In rodents, the EAE scoring is equivalent to the Expanded Disability Status Scale (EDSS) in the MS patients, which evaluates disease progression [26]. EAE scoring also associates with CNS injury, as well as areas of inflammatory lesions [27–29]. In accordance with earlier data [28, 30] untreated EAE groups in this study showed the highest EAE score on day 17 after EAE induction. Exercise without treatment did not succeed to reduce the acuteness of EAE, where no significant differences were observed between sedentary and exercised untreated EAE rats. Though the EAE score was halved in this study with mitoxantrone treatment on day 17 compared to EAE values without exercise, surprisingly, exercise has worsened the mitoxantrone EAE score to mimic the untreated EAE group. On the contrary, regular exercise of EAE rats treated with mitoxantrone clearly improved the motor performance of the EAE rats on day 17, as expected from earlier studies, which showed that exercise training improved MS performance [27, 30]. The rotarod results of untreated EAE exercised rats has matched previous reports [28, 31]; where the motor performance markedly decreased 13 days post EAE induction and thereafter.

As rotarod performance correlated with central inflammatory lesions and demyelinated motor nerves in the CNS [27], accordingly, physical exercise together with mitoxantrone treatment in this study shows a possible enhancement of motor activity in MS patients, however, worsening of the EAE score confuses this issue. In 2016, van den Berg and his colleagues [27] indicated that compared to the neurological/clinical scoring system, motor performance measured by the rotarod test was more objective and quantitative. They also reported that this motor performance test strongly correlated to the surface area of inflammatory lesions in the motor systems. Such data emphasize that the rotaroad test could be a better indication for the ability of mitoxantrone to resolve EAE motor symptoms, an effect confirmed by the reduction of the inflammatory score assessed microscopically in the brain stem to resolve the puzzle about the aberrant contradictory behavioral observations in the mitoxantrone exercised EAE rats. After finalizing our work, other exercise protocols, like swimming or voluntary running wheel, have been recently introduced to show the effectiveness in several EAE models [32, 33]; these tests hence, could be considered an alternative option with drug treatment, which needs further inspection.

The aberrant enhanced EAE score of mitoxantrone exercised rats could be clarified by the present unchanged demyelination score in LFB stained sections versus EX-EAE alone. The histopathological changes and LFB staining confirmed the destructive effect of EAE to the neurons, where both inflammatory and demyelinating scores were elevated, effects that matched the results of Zhang et al. [21]. Adding to the earlier data in this study, treatment with mitoxantrone has shown an obvious neuronal protective effect, partly by diminishing inflammation and improving neuronal remyelination markers with or without exercise. Nonetheless, the latter was not reflected on demyelination score though it showed a tendency to reduce it without reaching a significant level.

The potential anti-inflammatory effect of mitoxantrone has been evaluated here by measuring the changes in the pro-inflammatory cytokine, TNF-α, which is related to the pathogenesis of MS. Elevated TNF-α levels were associated with disease severity [34, 35], as well as myelin and axonal damage [34, 36]. It has also been considered as one of the causes of delayed remyelination [34]. As reported by Bielekova and Martin, [37] measuring cytokine levels in the CSF, but not peripheral blood, could give a better indication of MS severity, a fact that was formerly proven by Villarroya et al. [38]. In the current study, administration of mitoxantrone did not alter the elevating effect of EAE on TNF-α, neither in exercised groups nor sedentary ones. As noted by Gbadamosi et al.[39], short term treatment of MS patients with mitoxantrone did not alter TNF-α level, a finding that supports the present result to verify that this cytokine does not play a role in mitoxantrone-mediated improvements.

Similar to TNF-α, IL-6 affects demyelination, as well as inflammation [40, 41] and its altitude in CSF of MS patients is associated with the severity of the disease [40, 41]. Measuring IL-6 in the CSF was better correlated to disease severity than TNF-α, where mitoxantrone resulted in a decrease of its CSF levels compared to EAE rats whether sedentary or exercised to explain the present reduced inflammatory and/or demyelination scores with the treatment. Exercise training alone did not have an additional value to the effect of mitoxantrone on IL-6 levels. As a result of less inflammatory infiltrates and cytokines, one can assume that the attenuated EAE course was not only due to increased remyelination, but rather a protection from demyelination, especially considering that the animals were sacrificed at the peak of disease and not in the recovery phase.

Apart from the documented elevation of inflammatory markers in MS, this disease is also correlated with a prominent decline in the neurotrophic factor BDNF in serum of MS patients [34] and EAE animal models [41, 42], facts that support the current findings. In our study, EAE induction has shown an evident decrease in the CSF level of BDNF, whereas treatment with mitoxantrone has shown a clear improvement in BDNF levels in both exercised and sedentary rats. This in turn can partly explain the neuroprotective effect of mitoxantrone as confirmed in the histopathology examination, as well LFB staining and in earlier studies [41, 43]. Unfortunately, exercise training did not have any added value on the BDNF level compared to mitoxantrone sedentary group.

Another factor contributing to neuronal survival is the T cell population. In a previous study, Dombrowski et al. [44], has reported that Treg cells are able to promote myelination and remyelination and its transcriptional factor, Foxp3, is a good indicator of Tregs activity [21, 45]. Nevertheless, this point is debatable, where other studies revealed that Tregs activity is unrelated to Foxp3 count [46, 47]. Unexpectedly, our results showed a marked increase in the Foxp3 positive T lymphocytes in the EAE untreated group relative to normal control group, an effect that was even more evident in the sedentary rats over the exercised ones. This finding can be attributed to the aptitude of mitoxantrone to improve myelination, which did not necessitate the recruitment of Tregs. This notion can be supported by the high expression of Foxp3 in the untreated model possibly as a kind of body compensatory mechanism to control the acute period of the disease as mentioned earlier by Irony-Tur-Sinai et al. [45]. On the other hand, studies that showed an accumulation of Tregs in EAE brain have stated that this was an unsuccessful attempt to counteract local autoimmunity [48], meaning that, despite their brain recruitment, their function is impaired during EAE. Treatment with mitoxantrone, however, caused a sharp decrease in the number of Foxp3 T lymphocytes in the sedentary group to correspond to the results reported by Hanes et al. [49] and D’Arena et al. [50], pointing to the efficacy of mitoxantrone in controlling the acuteness of the disease. Although the exercise did not add any beneficial effect to the mitoxantrone treated groups, yet its impact was obvious in the exercised EAE rats. This group showed that exercise training decreased the overall immunoreactivity in the brain stem, as manifested in the decreased Foxp3 level in exercised EAE rats compared to its sedentary counterpart.

We also evaluated whether mitoxantrone mediates its effect by preserving neuronal cell survival via examining the immunostaining of caspase-3, which is implicated in cell death, axonal damage, neuronal apoptosis and inflammation in EAE [51, 52]. As observed in this study and previously reported, EAE rats showed a prominent increase in caspase-3 positive cells, making it an important target to decrease the axonal-damage and degeneration in MS using specific caspase-3 inhibitors [51]. Post-treatment with mitoxantrone succeeded to decrease the amount of caspase-3 positive cells, effect that was further reduced in the exercised rats receiving mitoxantrone relative to their sedentary counterparts to highlight the role of exercise in protecting neurons against apoptosis. Such influence of exercise in reducing caspase-3 expression has been earlier described in EAE mice hippocampus [53].

The effect of mitoxantrone with/without exercise on the CSF level of the anti-apoptotic biomarker Bcl-2 and the pro-apoptotic biomarker Bax were also evaluated. While sedentary/ exercised untreated EAE groups have shown a sharp decline in Bcl-2 levels, they have increased the Bax levels to concur with earlier findings in MS patients and EAE animal models [54, 55]. The effect of exercise in minimizing cell death was evidenced in results of these markers, comparing exercised with the sedentary values in all treated and untreated groups, such effects have been reported earlier by Kim et al. [53] in the EAE mice hippocampus. Surprisingly, the results of mitoxantrone pointed to its apoptotic potential in this model, where it failed to oppose the EAE effect on Bcl-2 and even worsened the EAE effect on the apoptotic marker Bax. Remarkably, mitoxantrone decreased the Bcl-2/Bax ratio in the CSF confirming its well-known apoptotic pathway [56, 57]; however, it simultaneously decreased caspase-3 level in the spinal cord. Since Bcl-2 and Bax are mainly related to the intrinsic type of apoptosis, in which the mitochondrial function plays a critical role, then this unexpected effect of mitoxantrone could be owed to its ability to cause mitochondrial energetic imbalance that results in ATP decrease, beside other mitochondrial dysfunctions [58]**.** However, the role of mitochondrial topoisomerase, a critical mitochondrial integrity enzyme, on the effect of mitoxantrone cannot be excluded being a DNA topoisomerase inhibitor [59]. The apoptotic potential of mitoxantrone has also been documented previously [60, 61]. According to this notion, one can speculate that the inhibited caspase-3 may be due to the extrinsic apoptotic event, although TNF-α, which is one element of this pathway, was also not altered by mitoxantrone, a point that needs thorough investigation.

As a further confirmation for the potential toxic effect of mitoxantrone, rats treated with mitoxantrone revealed blue discoloration of their testis and the porphyrin spots around their eyes. Indeed, mitoxantrone pharmacokinetics profile might explain these features, as Batra et al. [62] reported that multiple doses of mitoxantrone in animals showed an extensive distribution into tissues and a slow elimination to clarify the already proven toxicity of mitoxantrone [63]. A previous study by Williams [64] proved that porphyrin‐pigments and lipid‐laden tears were predominantly produced from the Harderian gland as a normal phenomenon, but increases in the case of chromodacryorrhoea as a warning sign of potentially severe systemic disease or physiologic stresses.

## Conclusions

In conclusion, mitoxantrone has partially minimized the EAE severity, via enhancing remyelination, protecting from demyelination, augmenting the neurotrophic factor BDNF and reducing IL-6. However, its anti-apoptotic effect needs further investigation; while it succeeded to abate caspase-3 efficiently it failed to correct Bcl2 and Bax. On the other hand, exercise training alone did not add a significant value to most studied parameters, except for a reduction in the brain stem Foxp3 immunoreactivity. To our knowledge, this is the first study examining the effect of exercise with mitoxantrone treatment on the neuronal function and disease management in EAE model. Whether physical exercise improves or deteriorates treatment of MS with mitoxantrone, still needs further investigation and validation with more attention directed towards the apoptotic cascade. A longer observation period of the effect of exercise with mitoxantrone could be also required.

## Supplementary Information

**Additional file 1.** Changes in motor performance of exercised rats during the 14 training days before induction.

## Data Availability

All data generated or analysed during this study are included in this published article.

## Abbreviations

CSF: Cerebrospinal fluid
CN: Control
EAE: Experimental Autoimmune Encephalomyelitis
EX: Exercised rats
IL: Interleukin
LFB: Luxol fast blue
MS: Multiple sclerosis
MT: Mitoxantrone
SED: Sedentary rats
TNF-α: Tumor necrosis factor-α
