# Supplementary material for: Does physical exercise improve or deteriorate treatment of multiple sclerosis with mitoxantrone? Experimental autoimmune encephalomyelitis study in rats
Source: BMC Neurosci. 2022 Mar 5;23:11. doi: 10.1186/s12868-022-00692-1 (PMC8897955; doi:10.1186/s12868-022-00692-1)
Supplement: Supplementary file 1 — Additional file 1. Changes in motor performance of exercised rats during the 14 training days before induction. [file 12868_2022_692_MOESM1_ESM.docx]

| **Motor performance (sec)** | **Days** | **Motor performance (sec)** | **Days** |
| --- | --- | --- | --- |
| 1793±6.667 ^€^ | **Day 8** | 1321±95.69 | **Day 1** |
| 1800±0^€^ | **Day 9** | 1562±77.57 ^€^ | **Day 2** |
| 1800±0^€^ | **Day 10** | 1687±46.95 ^€^ | **Day 3** |
| 1800±0^€^ | **Day 11** | 1708±52.67^€^ | **Day 4** |
| 1800±0^€^ | **Day 12** | 1745±31.10^€^ | **Day 5** |
| 1800±0^€^ | **Day 13** | 1769±31.48^€^ | **Day 6** |
| 1800±0^€^ | **Day 14** | 1776±21.41^€^ | **Day 7** |

**Changes in motor performance of exercised rats during the 14 training days before induction**

Day 1 is the first day of training and day 14 is the last day of training before induction. Values are presented as mean ± S.D (n=6). Data were analyzed using paired t- test (*p*<0.05).As compared with (€) day 1.
